# Você Vai Avisá-los Quando Estiverem Errados? Viés de Automação e Inteligência Artificial na Medicina

**DOI:** 10.36660/abc.20250300

**Published:** 2025-06-26

**Authors:** Marcio Sommer Bittencourt

**Affiliations:** 1 University of Pittsburgh Department of Medicine Division of Cardiology Pittsburgh PA EUA Division of Cardiology, Department of Medicine, University of Pittsburgh, Pittsburgh, PA – EUA

**Keywords:** Inteligência Artificial, Cardiologia, Viés

O uso de ferramentas de suporte à decisão e de automação na prática médica não é algo recente. As primeiras ferramentas utilizavam modelos de regressão simples para reduzir a heterogeneidade e oferecer um cuidado mais estruturado. Na cardiologia, exemplos iniciais de ferramentas de suporte à decisão incluíram o escore de risco de Framingham para estratificação de risco na prevenção primária e os ajustes automatizados de doses para heparina ou varfarina. Desde então, os processos de automação tornaram-se mais sofisticados e atualmente estão amplamente disseminados em diversos aspectos rotineiros da prática médica. Na cardiologia, incluem interpretações preliminares automatizadas de traçados de eletrocardiograma (ECG), bem como medições e análises de imagens automatizadas em ecocardiografia, medicina nuclear e ressonância magnética cardíaca. Outros sistemas automatizados de suporte à decisão clínica auxiliam na redução de erros de prescrição, alertando os profissionais de saúde sobre interações medicamentosas ou dosagens incorretas, por exemplo. Com os avanços recentes da inteligência artificial (IA), as expectativas quanto ao futuro dessas ferramentas cresceram de forma expressiva. No entanto, as possíveis implicações negativas têm recebido consideravelmente menos atenção.

Os sistemas de suporte à decisão podem aprimorar a tomada de decisão médica e os desfechos dos pacientes.^1^ No entanto, esses sistemas não são infalíveis e podem gerar resultados incorretos. A maioria dos estudos que avaliam seu desempenho baseia-se em métricas médicas convencionais, como sensibilidade, especificidade, acurácia e área sob a curva *receiver operating characteristic*. Contudo, é ainda mais importante avaliar o impacto dessas ferramentas no mundo real, especialmente considerando a forma como os profissionais interagem com elas. Essa questão torna-se particularmente relevante à luz do Regulamento de Inteligência Artificial da União Europeia, que exige supervisão humana para sistemas de IA considerados de alto risco. O tema adquire ainda mais relevância com o uso disseminado de *chatbots* de IA generativa, uma vez que a atual geração de ferramentas complexas de IA tem contribuído para uma tendência crescente de dependência excessiva de processos de automação imperfeitos.

Essa dependência excessiva pode ser compreendida a partir de dois conceitos relacionados, porém distintos: viés de automação e complacência com a automação. O viés de automação refere-se à tendência humana de confiar nos resultados fornecidos por ferramentas automatizadas em detrimento de informações não automatizadas, como o julgamento pessoal ou a contribuição de outros indivíduos. A complacência com a automação, por sua vez, é uma manifestação diferente do mesmo fenômeno. Ela descreve uma sensação de confiança ou satisfação excessiva em relação aos resultados de sistemas automatizados, o que leva à redução da vigilância e à aceitação acrítica de seus resultados.^2^

Um exemplo simples na cardiologia envolve o uso de softwares de pré-leitura de ECGs. Devido à dependência excessiva dos processos automatizados, o clínico pode sentir-se compelido a incluir no laudo conclusões que não consegue identificar com segurança nos traçados, pressupondo que o software seja mais preciso do que sua própria interpretação. Alternativamente, o profissional pode tornar-se tão injustificadamente dependente do sistema que deixa de avaliar adequadamente os segmentos ST em casos de supra de ST — a menos que essas alterações sejam sinalizadas pelo próprio sistema. Esse comportamento reflete uma redução da vigilância decorrente da confiança excessiva na ferramenta de suporte à decisão, associada a uma sensação inadequada de segurança na interpretação automatizada. As consequências desses erros podem ser significativas. Um estudo de 2004 sobre ferramentas de interpretação automatizada de ECG constatou que mais de dois terços dos traçados diagnosticados incorretamente como fibrilação atrial não foram corrigidos pelo médico que interpretava o exame. Isso resultou no uso inadequado de antiarrítmicos e anticoagulação em um terço dos casos com diagnóstico automatizado incorreto.^3,4^

Embora seja necessário reconhecer que as ferramentas de automação evoluíram desde 2004, sistemas com maior acurácia podem, paradoxalmente, aumentar o viés de automação, uma vez que sua suposta confiabilidade pode levar a uma dependência ainda maior. Além disso, uma extensa literatura indica que o risco de viés de automação aumenta à medida que a tarefa se torna mais difícil e complexa.^5^ Com a contínua evolução da IA, as ferramentas de suporte estão se tornando mais complexas e sendo aplicadas a tarefas cada vez mais desafiadoras. Portanto, é fundamental que os profissionais de saúde compreendam as potenciais consequências do viés de automação e da complacência com a automação, bem como as estratégias disponíveis para mitigar esses riscos.

Os principais fatores que contribuem para o viés de automação incluem a suposição de que máquinas e sistemas automatizados são infalíveis. Além disso, a maioria dos sistemas automatizados apresenta maior facilidade de uso em comparação com métodos tradicionais (p.ex., é mais simples relatar a frequência cardíaca calculada pelo software em um ECG do que calculá-la manualmente), e os seres humanos tendem naturalmente a preferir a eficiência. Por fim, temos a tendência de confiar mais em sistemas que não compreendemos completamente — uma questão especialmente relevante para a atual geração de ferramentas de automação baseadas em IA, cujos parâmetros subjacentes frequentemente são desconhecidos pelo usuário.

Para mitigar as consequências do viés de automação, são necessárias intervenções multifacetadas. Essas intervenções devem abranger o usuário da ferramenta de automação, o design da própria ferramenta e os processos organizacionais que influenciam seu uso. Do ponto de vista do usuário, estratégias abrangentes de treinamento e educação são fundamentais para aumentar a conscientização sobre as limitações e potenciais erros dos sistemas automatizados. Isso inclui tanto a educação geral sobre ferramentas de automação baseadas em IA quanto treinamentos específicos de cada ferramenta, a fim de ajudar os usuários a reconhecer quando uma determinada ferramenta tende a apresentar bom — ou mau — desempenho. Esse treinamento direcionado pode fomentar um pensamento mais crítico, permitindo que os usuários avaliem e questionem melhor os resultados automatizados, reduzindo, assim, o risco de complacência com a automação.

Sob a perspectiva do design, é essencial garantir que as ferramentas sejam devidamente validadas, considerando o componente de interação humana. Mais do que relatar apenas métricas de desempenho, como acurácia, essas ferramentas devem também ser avaliadas com base em seu impacto no cuidado clínico após a implementação. As consequências dos erros podem ser mais relevantes do que sua frequência — por exemplo, deixar de identificar um infarto agudo do miocárdio com supradesnivelamento do segmento ST é muito mais crítico do que não detectar extrassístoles ventriculares. Compreender com que frequência os profissionais cometem erros por ação ou omissão ao utilizar essas ferramentas também é fundamental. Além disso, os desenvolvedores devem trabalhar em estreita colaboração com os usuários finais para obter feedback sobre o desempenho em situações reais e identificar oportunidades de melhoria. Fechar esse ciclo de feedback entre desenvolvedores e usuários é essencial para aprimorar tanto a qualidade dos processos automatizados quanto sua implementação bem-sucedida.

Por fim, as organizações de saúde devem estabelecer processos que garantam a implementação das ferramentas de automação em contextos adequados. Por exemplo, uma ferramenta de interpretação de ECG pode ser mais útil em um departamento de emergência remoto, sem a presença de um cardiologista, do que em um grande centro de cardiologia. As instituições precisam considerar o contexto em que cada ferramenta será utilizada, bem como os protocolos, fluxos de trabalho, treinamentos e certificações necessários, a fim de implementar essas ferramentas de forma eficaz, minimizando o risco de viés de automação.
