# Supplementary material for: Você Vai Avisá-los Quando Estiverem Errados? Viés de Automação e Inteligência Artificial na Medicina
Source: Arq Bras Cardiol. 2025 Jun 26;122(5):e20250300. [Article in Portuguese] doi: 10.36660/abc.20250300 (PMC12269908; doi:10.36660/abc.20250300)
Supplement: Translation

# Translation: Will You Tell Them When They Are Wrong? Automation Bias and Artificial Intelligence in Medicine

**DOI:** 10.36660/abc.20250300i

**Authors:** Marcio Sommer Bittencourt

**Affiliations:** 1 Pittsburgh PA USA Division of Cardiology, Department of Medicine, University of Pittsburgh, Pittsburgh, PA – USA

**Keywords:** Artificial Intelligence, Cardiology, Bias

The use of decision support tools and automation in medical practice is not new. Early tools employed simple regression models to reduce heterogeneity and offer more structured care. In cardiology, initial examples of decision support tools included the Framingham risk scores for primary prevention risk stratification and automated sliding scale dose adjustments for heparin or warfarin. Since then, automation processes have become more sophisticated and are now widespread in many routine aspects of medical practice. In cardiology, these include automated preliminary interpretations of electrocardiography (ECG) tracings as well as automated measurements and image analysis in echocardiography, nuclear medicine, and cardiac magnetic resonance imaging. Other automated clinical decision support systems help reduce prescribing errors by alerting clinicians to drug-drug interactions or incorrect dosages, for example. With recent advances in artificial intelligence (AI), expectations for the future of such tools have grown dramatically. Yet, the potential negative implications of these technologies have received considerably less attention.

Clinical decision support systems can improve medical decision-making and patient outcomes.^1^ However, these systems are not flawless and may produce incorrect outputs. Most studies assessing their performance rely on conventional medical metrics such as sensitivity, specificity, accuracy, and area under the receiver operating characteristic curve. Yet, it is far more important to evaluate the real-world impact of these tools, particularly in light of how clinicians interact with them. This is especially relevant given that the European Union Artificial Intelligence Act mandates human oversight for high-risk AI systems. The issue becomes even more critical with the widespread use of generative AI chatbots, as the current generation of complex AI tools has led to a growing trend of overreliance on imperfect automation.

This overreliance can be understood through two related but distinct concepts: automation bias and automation complacency. Automation bias refers to the human tendency to rely on outputs from automated tools rather than on non-automated cues, such as personal judgment or input from other individuals. Automation complacency, on the other hand, is a different expression of the same phenomenon. It describes a sense of undue confidence or satisfaction with the output of automated systems, which leads to reduced vigilance and the uncritical acceptance of their results.^2^

A simple example in cardiology involves the use of pre-reading software for ECGs. Due to overreliance on automated processes, a clinician may feel compelled to include in the report conclusions they cannot confidently identify in the tracings, assuming the software is more accurate than their own interpretation. Alternatively, the clinician may become so unjustifiably dependent on the software that they fail to assess ST segments properly in cases of ST elevation — unless those abnormalities are flagged by the system. This reflects reduced vigilance resulting from overreliance on the decision support tool and a misplaced sense of confidence in the automated interpretation. The consequences of such errors can be significant. A 2004 study on automated ECG interpretation tools found that more than two-thirds of tracings incorrectly diagnosed as atrial fibrillation were not corrected by the interpreting physician. This led to inappropriate use of antiarrhythmic drugs and anticoagulation in one-third of the cases with incorrect automated diagnoses.^3,4^

While it is fair to acknowledge that automation tools have improved since 2004, higher-accuracy systems may increase automation bias since their perceived reliability can lead to greater overreliance. Furthermore, a substantial body of literature indicates that the risk of automation bias rises with increasing task difficulty and complexity.^5^ As AI continues to evolve, supporting tools are becoming more complex and are being applied to increasingly challenging tasks. Therefore, clinicians should understand the potential consequences of automation bias and automation complacency as well as the strategies available to mitigate these risks.

The primary drivers of automation bias include the human assumption that machines and automated systems are infallible. In addition, most automated systems are easier to use than traditional methods (e.g., it is simpler to report the software-calculated heart rate in an ECG than to calculate it manually), and humans naturally prefer efficiency. Finally, we tend to rely more heavily on systems we do not fully understand — an issue particularly relevant to the current generation of AI-based automation tools, where the underlying parameters are often unknown to the user.

To mitigate the consequences of automation bias, multifaceted interventions are required. These interventions must address the user of the automation tool, the technical design of the tool itself, and the organizational processes that influence its use. From the user's perspective, comprehensive training and educational strategies are essential to raise awareness of the limitations and potential errors of automated systems. This includes both general education about AI-based automation tools and tool-specific training to help users recognize when a particular tool is likely to perform well — or poorly. Such targeted training can foster more critical thinking, enabling users to better assess and question automated outputs, thereby reducing the risk of automation complacency.

From a design perspective, it is essential to ensure that tools are properly validated, considering the human interaction component. Rather than reporting only performance metrics such as accuracy, these tools must also be evaluated based on their impact on clinical care once implemented. The consequences of errors may be more important than their frequency — for example, missing a case of ST-elevation myocardial infarction is far more critical than overlooking premature ventricular contractions. Understanding how often clinicians commit errors of commission or omission when using these tools is also key. Additionally, developers must work closely with end users to gather feedback on real-world performance and identify opportunities for improvement. Closing this feedback loop between developers and users is vital to enhancing both the quality of automated processes and their successful implementation.

Finally, health care organizations must establish processes that ensure automation tools are implemented in appropriate settings. For example, an ECG interpretation tool may be more valuable in a remote emergency department without an on-site cardiologist than in a large cardiology center. Institutions must consider the context in which each tool will be used, along with the necessary protocols, workflows, training, and certification, in order to implement these tools effectively while minimizing the risk of automation bias.
